# The Characteristics of Natural Killer Cells in Chronic Hepatitis B Patients Who Received PEGylated-Interferon versus Entecavir Therapy

**DOI:** 10.1155/2021/2178143

**Published:** 2021-01-25

**Authors:** Weihua Cao, Minghui Li, Lu Zhang, Yao Lu, Shuling Wu, Ge Shen, Min Chang, Ruyu Liu, Yuanjiao Gao, Hongxiao Hao, Leiping Hu, Wei Yi, Calvin Q. Pan, Yao Xie

**Affiliations:** ^1^Department of Hepatology Division 2, Beijing Ditan Hospital, Capital Medical University, No. 8 Jing Shun East Street, Chaoyang, Beijing 100015, China; ^2^Department of Infectious Diseases, Miyun Teaching Hospital, Capital Medical University, Beijing 101500, China; ^3^Department of Hepatology Division 2, Peking University Ditan Teaching Hospital, No. 8 Jing Shun East Street, Chaoyang, Beijing 100015, China; ^4^Department of Obstetrics and Gynecology, Beijing Ditan Hospital, Capital Medical University, No. 8 Jing Shun East Street, Chaoyang District, Beijing 100015, China; ^5^Center of Liver Diseases, Beijing Ditan Hospital, Capital Medical University, Beijing 100015, China; ^6^Division of Gastroenterology and Hepatology, Department of Medicine, NYU Langone Health, New York University School of Medicine, New York, NY, USA

## Abstract

**Background:**

To explore the role of natural killer (NK) cells in the process of hepatitis B virus (HBV) clearance and whether their phenotype is related to antiviral treatment outcome in chronic hepatitis B (CHB) patients.

**Method:**

We performed a single-center prospective cohort study to analyze changes of NK cells at weeks 12 and 24 from baseline in CHB patients who received PEGylated-interferon- (PEG-IFN-) *α*-2a versus entecavir. The frequencies of NK, CD56^bright^, CD56^dim^, IFNAR2^+^, NKp46^+^, NKp46^bright^, and NKp46^dim^ NK cells and mean fluorescence intensity (MFI) of receptors NKp46 and IFNAR2 on the surface of NK cells were measured. Subgroup analyses were performed by comparing treatment responders versus nonresponders with aforementioned parameters in each group.

**Results:**

In PEG-IFN-*α*-treated patients, posttreatment CD56^bright^ NK cell frequency increased, but CD56^dim^ NK cell frequency decreased. Additionally, receptor NKp46 and IFNAR2 expression enhanced. In entecavir-treated patients, although NK cell frequency increased, CD56^bright^ and CD56^dim^ NK cell frequencies and IFNAR2 expression did not differ between baseline and posttreatment. In subgroup analyses, posttreatment CD56^bright^ NK cell frequency and IFNAR2 expression significantly increased in PEG-IFN-*α* responders from baseline, while changes were absent in PEG-IFN-*α* nonresponders and entecavir treatment responders. Among patients with HBV viremia after entecavir therapy, NK cell frequency significantly increased, whereas NKp46^bright^ and IFNAR2^+^ NK frequency and IFNAR2 MFI significantly decreased at 12 and 24 weeks from baseline.

**Conclusions:**

In CHB patients, PEG-IFN-*α* treatment significantly enhanced NK cell frequency and function when compared to entacavir. Positive treatment responses to either interferon or entecavir were associated with NK cell function improvement. This trial is registered with clinical trial registration no. NCT03208998.

## 1. Introduction

In hepatitis B virus (HBV) infection, hepatocellular injury or necrosis has been suggested as results from the host's immune response to the viruses, rather than direct effects of HBV infection [1]. HBV proteins are a major factor in determining virus clearance, chronic infection, and liver cell damage [1]. Natural killer (NK) cells, a key component of innate immunity, not only play a very important role in clearing the virus but also act a pivotal part in immune defense. NK cells also serve in immune surveillance and augmentation for other pathways of immune responses, resulting in liver damage while attempting the elimination of virus infection [2]. NK cells consist of two classic subpopulations, CD56^bright^ and CD56^dim^ cells. As the majority of the NK cell population, CD56^dim^ NK cells are identified as mature NK cells, whereas CD56^bright^ NK cells are the minority and deemed to be in the early stage of maturation [3]. It has been suggested that CD56^dim^ NK cells are mainly responsible for killing the virus-infected cells directly. In contrast, CD56^bright^ NK cells play a role in activating the adaptive immune system by secreting immunomodulatory cytokines such as IFN-*γ*, TNF-*α*, TGF-*β*, and IL-10 [4–6]. The cytotoxicity of NK cells is regulated by inhibitory and activating receptors to ensure proper activation against virus while preventing abnormal attacks to hepatocyte [7]. Activating receptor NKp46 on NK cells surface is a key receptor that initiates the killing activity of NK cells. Activated NK cells kill target cells by releasing cytotoxic particles such as perforin and granzyme. Numerous studies have demonstrated that activated NK cells cause liver damage while clearing hepatitis B virus [2, 8, 9]. Hepatitis B surface antigen (HBsAg) can directly inhibit NK cell function [10, 11]. HBeAg also downregulates IFN-*γ* production by NK cells [12]. In acute hepatitis B, Zhao et al. reported that the cytotoxicity of NK cells and the secretion of IFN-*γ* from NK cells increased, accompanied by the upregulation of the expression of activating receptor NKp46 and increasing the frequency of NK cells, in particular CD56^bright^ NK cell subpopulation [13]. However, the virus-specific CD8^+^ T cells demonstrate deficient response and functional depletion in CHB infection, which may contribute to the long-term persistent infection of HBV [13]. Zhao et al. also suggested that NK cells show an important role in the depletion of virus-specific CD8^+^ T cells [13]. In HCV and HIV infection, activated NK cells could clear CD4^+^ T cells and subsequently affect the function of CD8^+^ T cells [14].

Previous studies observed that NK cells presented with selectively functional deficiency [5] and relatively conservative or even unchanged cytotoxicity in patients with CHB, resulted in the suppression of the production of cytokines IFN-*γ* and TNF-*α* in clearance of hepatitis B virus [15–17]. The inhibition of viral replication through antiviral therapy has been suggested to durable clinical endpoints by restoring the NK cell activities including the production of IFN-*γ* [16]. However, data on the dynamic changes of NK cell frequency and function during antiviral therapy are limited.

Currently, PEG-IFN-*α* and nucleoside analogues are main streams of antiviral drugs for CHB. Although a few studies have observed changes of receptors NKp46 and IFNAR2 expression on NK cells during interferon treatment in CHB patients [18–22], the correlation between other NK cell functions or frequency of subpopulation of NK cells and HBV clearance remains unclear. Our study was to further characterize the dynamic changes in NK frequency and function during PEG-IFN or entecavir (ETV) therapy and assess the differences in between treatment responder versus nonresponder in each therapy.

## 2. Materials and Methods

### 2.1. Patients

This was a single-center, nonrandomized, parallel, prospective cohort study. Approximate consecutive 90 HBeAg-positive CHB patients were planned to be enrolled from October 2014 to October 2017. The major inclusion criteria were the following: HBsAg and HBeAg positivity for at least 6 months, detectable HBV DNA and abnormal ALT levels lasting for more than 3 months, or liver biopsy showing >stage 2 inflammation or fibrosis when the ALT were normal [23]. The exclusion criteria were composed of coinfection with other viruses including HCV, HDV, and HIV; syphilis antibody positive; co-exist other liver diseases including alcoholic liver disease, metabolic liver disease, fatty liver, drug induce liver injury, and autoimmune liver disease and complication of cirrhosis or liver cancer. All patients were treatment naïve, met the treatment criteria, anticipated to start on one of the aforementioned two therapies, were willing to provide consent, and were compliant with follow-up schedules. The institutional ethic review committee approved the study. All procedures were performed after obtaining patients' informed consent.

### 2.2. Study Design

90 HBeAg-positive CHB patients were planned to be enrolled into two groups with 40-45 in each, i.e., PEG-IFN group and entecavir group. After enrollment, the management of patients was based on the standard of care in our center as the following: treatment patients who received PEG-IFN therapy were given subcutaneous injection of PEG-IFN-*α* 180 *μ*g/weekly for 48 weeks with monitoring visits every 4 weeks, while some patients were treated with entecavir (ETV) at 0.5 mg/day for more than 48 weeks with monitoring visits every 12 weeks. The study specimens included collecting serum for measurement of HBsAg/anti-HBs levels, HBeAg/anti-HBe levels, and HBV DNA loads before treatment and during treatment at weeks 12, 24, 36, and 48; collecting blood for hematological and biochemical tests every 4 to 12 weeks based on the therapy they received; and, lastly, blood samples for NK cell frequency and function measurement before initiating the treatment and at weeks 12 and 24 for both groups.

PEG-IFN treated CHB patients were further divided into two groups for subgroup analyses, i.e., response group and nonresponse group based on the decline of HBsAg levels more than 60% from the baseline. Results of logistic regression analysis showed that the HBsAg decline was independently associated with the frequency of NKp46^bright^ NK cells at baseline and at treatment week 12 during early treatment with PEG-IFN-*α*. The decline of HBsAg levels above 60% was defined as HBsAg response, and the decline of HBsAg level below 60% was considered HBsAg nonresponse. In CHB patients treated with entecavir, patients were further subgrouped into responders and nonresponders based on the status of achieving undetectable levels of HBV DNA at 48 weeks of treatment.

Primary analyses were performed for the comparison of NK cells and their subpopulations in CHB patients who received PEGylated-interferon- (PEG-IFN-) *α*-2a versus entecavir, which included changes at weeks 12 and 24 of the therapy in frequencies of NK, CD56^bright^ NK, CD56^dim^ NK, IFNAR2^+^ NK, NKp46^+^ NK, NKp46^bright^ NK, and NKp46^dim^ NK cells in patients' peripheral blood from baseline. Secondary analyses were the subgroup comparison of the aforementioned parameter changes of NK cells at weeks 12 and 24 between treatment responders and nonresponders. The definition of responders was follows: patients who achieved >60% of decline at HBsAg titers with 48 weeks of PEG-IFN therapy were considered responders, or those who had undetectable levels of HBV DNA with 48 weeks of entecavir therapy were defined as responders. Other assessments included the relationship between the frequency or function of NK cells and baseline clinical parameters including levels of HBsAg titers, HBeAg titers, HBV DNA, and ALT.

### 2.3. Laboratory Assessment

Serum HBV DNA levels were measured by PCR quantitation with the Roche Cobas AmpliPrep/Cobas TaqMan96 and full automatic real-time fluorescent quantitative PCR detection reagent (Roche, Pleasanton, CA, USA), at the range of 20-10^8^ IU/mL. Serum HBsAg/anti-HB and HBeAg/anti-HBeAb levels were measured with the Abbott Architect i2000 Detection Reagent (Abbott Diagnostics, Abbott Park, IL, USA). The ALT levels were measured with the Hitachi 7600 full-automatic biochemical analyzer (Hitachi, Japan) with the upper limit of normal values of 40 U/L. All the tests were performed at the Clinical Laboratory Center of Beijing Ditan Hospital Affiliated to Capital Medical University in China.

### 2.4. Flow Cytometry of NK Cells

For the measurement of NK cell frequency and function, peripheral blood mononuclear cells (PBMCs) were isolated using standard density-gradient centrifugation and stained with fluorescent label-conjugated monoclonal antibodies (mAbs) for 20 min in the dark at room temperature: Mouse anti-Human-CD3-PerCP (Becton-Dickinson, USA; clone: SP34-2), Mouse anti-Human CD56-APC (Becton-Dickinson, USA clone: B159), anti-Human CD335 (NKp46)-PE (Biolegend, USA; clone: 9E2), and Anti-Human IFNAR2-FITC (Sino Biological, Beijing, China; clone: 07). After 20 min, 2 mL red blood cell lysis buffer (BD Biosciences) was mixed into the blood at stand for 5 min in the dark at room temperature. The supernatants were spun (1200 rpm/5 minutes) to remove cell debris. Then, cells were washed with 2 mL 1 × PBS and the supernatants were spun (1200 rpm/5 minutes) to remove cell debris.

In Supplementary Figure 1, cell phenotyping was performed using the FACSCalibur flow cytometer (BD Biosciences, New Jersey, USA). Specific steps were as follows: (1) circle the lymphocyte population in the human white blood cell population (Supplementary Figure 1A); (2) in lymphocyte population, NK cells were identified by the expression of CD56 and lack of CD3. According to the expression of CD56 on NK cells, CD3^−^CD56^+^ NK cells are divided into CD56^bright^ NK cells and CD56^dim^ NK cells (Supplementary Figure 1B); (3) NKp46^+^ NK cells were circled in NK cells, and NKp46^+^ NK cells were divided into NKp46^high^ NK cells and NKp46^dim^ NK cells (Supplementary Figure 1C); (4) Circle IFNAR2^+^ NK cells in NK cells (Supplementary Figure 1D). FlowJo 7.6.1 software was used to analyze the percentages of NK cells, CD56^dim^ and CD56^bright^ NK cells, NKp46-positive NK cells, and IFNAR2-positive NK cells, and MFI (mean fluorescence intensity) was used to define expression levels of NKp46 and IFNAR2. The percentage of NK cells within PBMCs was defined as NK frequency. The percentages of CD56^bright^, CD56^dim^, NKp46^+^, and IFNAR2^+^ subsets among total NK cells were defined as CD56^bright^, CD56^dim^, NKp46^+^, and IFNAR2^+^ NK cell frequency, respectively. The expressions of NKp46 and IFNAR2 receptors were for the functional measurement.

### 2.5. Statistical Analysis

Data were performed using SPSS for Windows software, version 21 (SPSS Inc., Chicago, IL, USA), and Prism software (GraphPad Software version 5.01). All data were presented as the median (Q1, Q3) or mean ± standard deviation (SD), as appropriate. Changes in the indexes at different time points were evaluated by repeated-measures analysis of variance. The association between variables was assessed by means of Pearson's correlation. A chi-square test for comparison between two independent samples. All analyses of significance were two-tailed, and *P* value of less than 0.05 was identified as statistical significance.

## 3. Results

### 3.1. Baseline Clinical Characteristics of Chronic Hepatitis B Patients

Among 100 patients who eligible for the study, 89 patients were enrolled with 59 males and a median age of 31 years old (range 20–53). Eleven patients were excluded from the study because six patients could not tolerate the side effects of interferon, and five patients did not take ETV regularly. Forty nine patients were treated with PEG-IFN and 40 received ETV, and all of these patients completed 48 weeks treatment. At baseline, there were no significant differences in serum HBV DNA loads, HBsAg and HBeAg concentrations, and ALT levels between the two groups (Table 1). During therapy, HBV DNA loads, HBsAg and HBeAg levels, and ALT levels decreased; HBsAg levels in the PEG-IFN group significantly decreased, compared with those in the ETV group (Table 2). In the PEG-IFN group, the rate of HBsAg response (the decline of HBsAg concentration more than 60% within 48 weeks) was 67.35% (33/49); HBV DNA loads of 45 patients were undetectable and HBeAg seroconversion occurred in 9 patients. In the ETV group, the rate of HBV DNA response (patients with undetectable HBV DNA load within 48 weeks) was 67.50% (27/40); HBeAg seroconversion occurred in 3 patients.

### 3.2. The Correlation between the Frequency and Function of NK Cells and HBV DNA/Antigen Levels before Treatment

**Figure 1:**
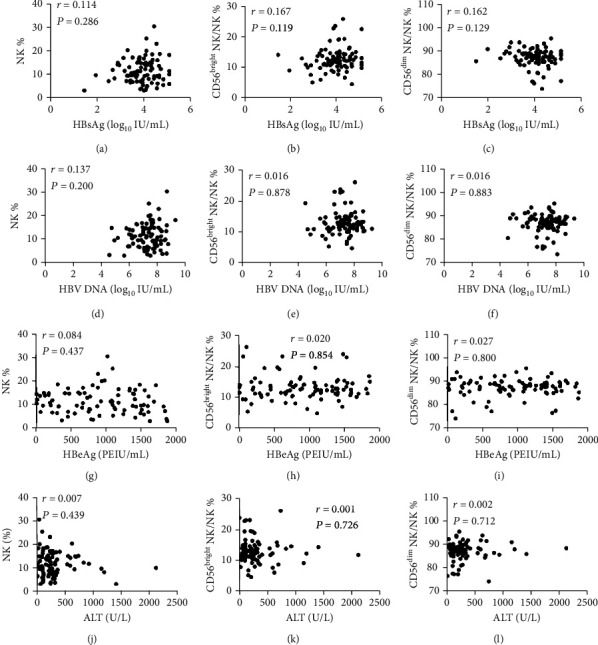
Relationship between the frequencies of NK cells and subpopulations of NK cells and clinical parameters at baseline. The correlation between HBsAg level and NK% (a), CD56^bright^ NK% (b), and CD56^dim^ NK% (c); the correlation between HBV DNA load and NK% (d), CD56^bright^ NK% (e), and CD56^dim^ NK% (f); the correlation between the level of HBeAg and NK% (g), CD56^bright^ NK% (h), and CD56^dim^ NK% (i); the correlation between ALT level and NK% (j), CD56^bright^ NK% (k), and CD56^dim^ NK% (l).

**Figure 2:**
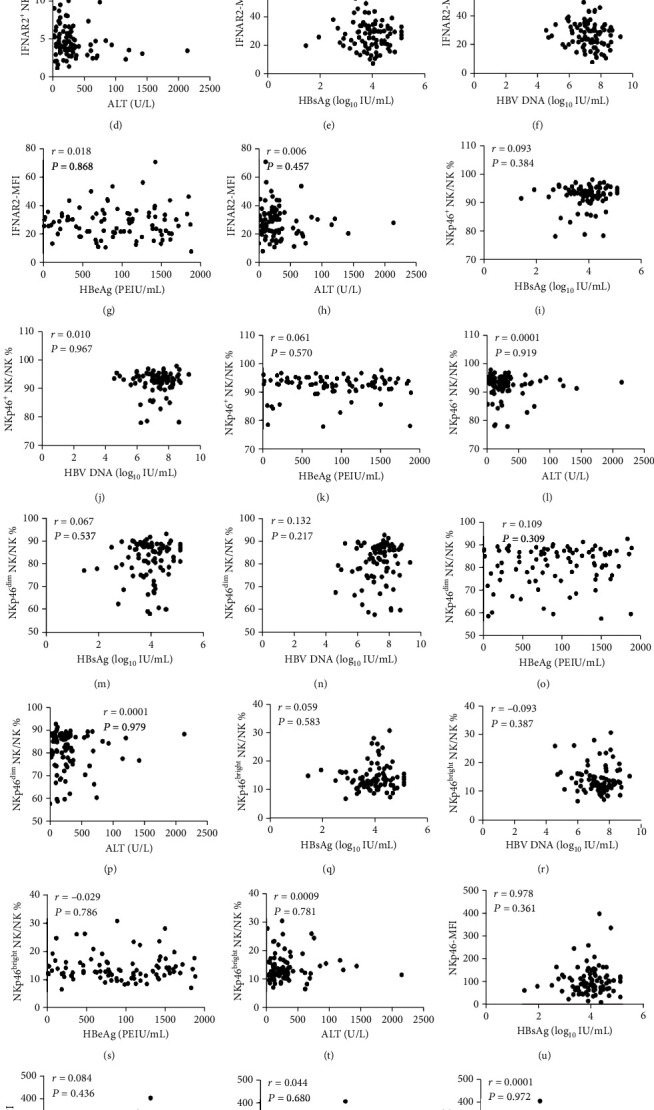
Relationship between the frequencies of IFNAR2^+^ NK and NKp46^+^ NK cells including their subpopulations and clinical parameters at baseline. The correlation between HBsAg level and IFNAR2^+^ NK% and HBsAg level (a), HBV DNA load (b), the level of HBeAg (c), and ALT level (d); the correlation between IFNAR2-MFI and HBsAg level (e), HBV DNA load (f), the level of HBeAg (g), and ALT level (h); the correlation between NKp46^+^ NK% and HBsAg level (i), HBV DNA load (j), the level of HBeAg (k), and ALT level (l); the correlation between NKp46^bright^ NK% and HBsAg level (m), HBV DNA load (n), the level of HBeAg (o), and ALT level (p); the correlation between NKp46^dim^ NK% and HBsAg level (q), HBV DNA load (r), the level of HBeAg (s), and ALT level (t); the correlation between NKp46-MFI and HBsAg level (u), HBV DNA load (v), the level of HBeAg (w), and ALT level (x).

At the baseline measurement, frequencies of NK cells, CD56^bright^ NK cells, and CD56^dim^ NK cells were not correlated to the levels of HBsAg, HBeAg, HBV DNA, and ALT as shown in Figure 1. Furthermore, we observed that the frequencies of NKp46^+^ NK cells and subpopulations of NKp46^bright^ and NKp46^dim^, and IFNAR2^+^ NK cells, did not correlate with the levels of HBV DNA, HBsAg, HBeAg, and ALT neither. In addition, we did not find associations between the MFIs of IFNAR2 and NKp46 and the levels of HBV DNA, HBsAg, HBeAg, and ALT (Figure 2).

### 3.3. NK Cell Frequencies and Function during the First 24 Weeks of PEG-IFN versus Entecavir Therapy

**Figure 3:**
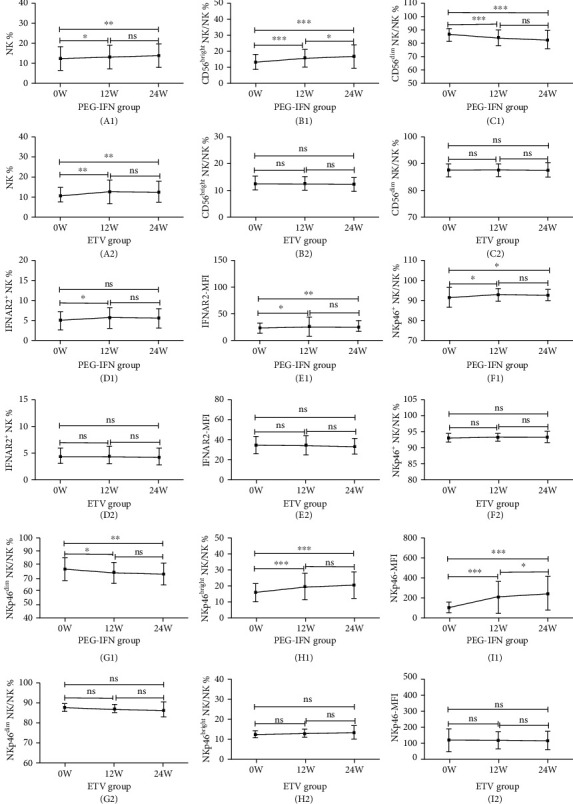
Tendencies of the frequencies and function of NK cells and their subpopulations before treatment and after therapy weeks 12 and 24 in the PEG-IFN and ETV group. The parameters include NK% (A1, A2), CD56^bright^ NK% (B1, B2), CD56^dim^ NK% (C1, C2), IFNAR2^+^ NK% (D1, D2), IFNAR2-MFI (E1, E2), NKp46^+^ NK% (F1, F2), NKp46^bright^ NK% (G1, G2), NKp46^dim^ NK% (H1, H2) cells, and NKp46-MFI (I1, I2). ^∗^*P* < 0.05; ^∗∗^*P* < 0.01; ^∗∗∗^*P* < 0.001; ns: not significant.

In the PEG-IFN treatment group, both the frequencies of NK, CD56^bright^ NK, IFNAR2^+^ NK, NKp46^+^ NK, and NKp46^bright^ NK cells and the MFIs of IFNAR2 and NKp46 significantly increased at treatment weeks 12 and 24 from baseline. In contrast, the frequencies of CD56^dim^ NK and NKp46^dim^ NK cells dramatically decreased at treatment weeks 12 and 24 in this group (Figure 3). In the ETV group, we observed that there were no significant differences when comparing measurements at the baseline, week 12, and week 24 in terms of frequencies of CD56^bright^ NK, CD56^dim^ NK, IFNAR2^+^ NK, NKp46^+^ NK, NKp46^bright^ NK, NKp46^dim^ NK cells, MFIs of IFNAR2, and NKp46, although the NK cell frequency increased markedly at treatment weeks 12 and 24 (Figure 3(A2)).

### 3.4. Subgroup Analyses on NK Cell Function

#### 3.4.1. NK Function and Treatment Response during PEG-IFN-*α* Therapy

**Figure 4:**
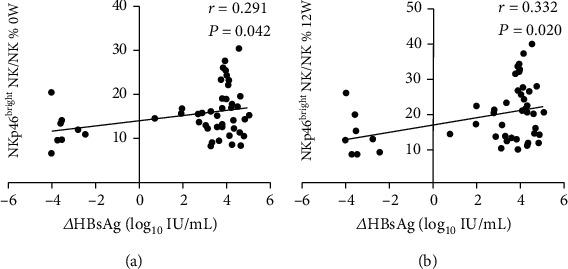
Correlation between the frequency of NKp46^bright^ NK and the decrease in HBsAg levels. The association of NKp46^bright^ NK frequency at baseline (a) and after PEG-IFN treatment for 12 weeks (b) and the decrease of HBsAg level after PEG-IFN treatment for 48 weeks.

**Figure 5:**
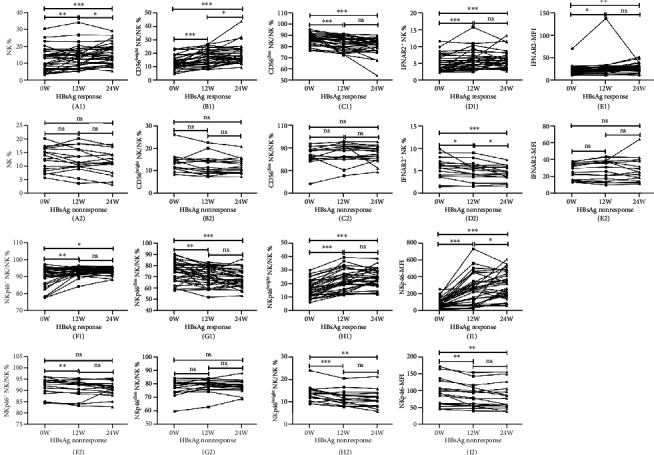
Tendencies of the frequency and function of NK cells and their subpopulations before PEG-IFN therapy and at treatment weeks 12 and 24 in the HBsAg response group versus nonresponse group. The parameters include NK% (A1, A2), CD56^bright^ NK% (B1, B2), CD56^dim^ NK% (C1, C2), IFNAR2^+^ NK% (D1, D2), IFNAR2-MFI (E1, E2), NKp46^+^ NK% (F1, F2), NKp46^bright^ NK% (G1, G2), NKp46^dim^ NK% (H1, H2) cells, and NKp46-MFI (I1, I2). ^∗^*P* < 0.05; ^∗∗^*P* < 0.01; ^∗∗∗^*P* < 0.001; ns: not significant.

Subgroup analyses were performed in each treatment group individually on NK cell frequencies and functions. In the PEG-IFN group, when comparing responders and nonresponders, the frequencies of NK and CD56^bright^ NK cells increased significantly at 12 and 24 weeks among responders. However, CD56^dim^ NK frequency decreased significantly at 12 and 24 weeks in responders. Although the changes in HBV DNA and HBeAg levels were not associated with NK cell function, we observed that the decline of HBsAg levels at week 48 of treatment was significantly associated with the NKp46^britht^ NK frequency at baseline and at 12 weeks of PEG-IFN therapy (Figure 4). In addition, the frequencies of IFNAR2^+^ NK, NKp46^+^ NK, and NKp46^bright^ NK cells and the MFIs of IFNAR2 and NKp46 increased significantly at week 12 and 24 weeks when compared with those at baseline, though NKp46^dim^ NK cell frequency decreased at those time points during INF treatment (Figure 5).

Among nonresponders, there were no significant changes in the frequencies of NK, CD56^bright^ NK, and CD56^dim^ NK cells from baseline to weeks 12 and 24, as shown in Figure 5. Other NK cell observation in this group included the following: the frequencies of IFNAR2^+^, NKp46^+^, and NKp46^bright^ NK cells, and NKp46 MFI decreased markedly from baseline to weeks 12 and 24; the NKp46^dim^ NK cell frequency and IFNAR2 MFI had no significant changes from baseline to weeks 12 and 24 (Figure 5).

#### 3.4.2. NK Function and Response to ETV Therapy

**Figure 6:**
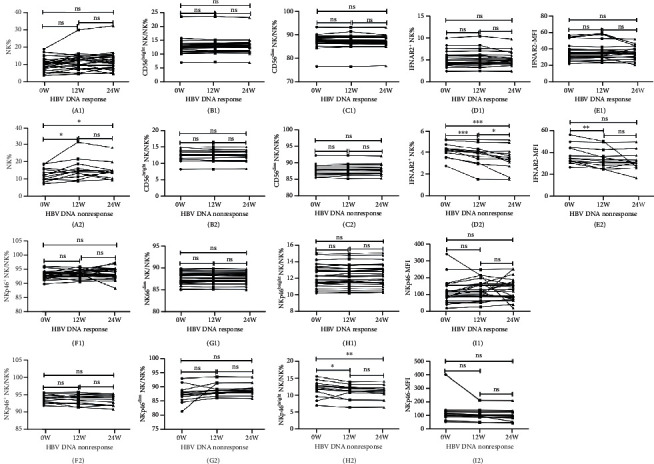
Tendencies of the frequency and function of NK cells and their subpopulations before ETV therapy and after treatment weeks 12 and 24 in the HBV DNA response versus nonresponse group. The parameters include NK% (A1, A2), CD56^bright^ NK% (B1, B2), CD56^dim^ NK% (C1, C2), IFNAR2^+^ NK% (D1, D2), IFNAR2-MFI (E1, E2), NKp46^+^ NK% (F1, F2), NKp46^bright^ NK% (G1, G2), NKp46^dim^ NK% (H1, H2) cells, and NKp46-MFI (I1, I2). ^∗^*P* < 0.05; ^∗∗^*P* < 0.01; ^∗∗∗^*P* < 0.001; ns: not significant.

In patients treated with ETV, the changes in HBsAg and HBeAg levels were not associated with the changes in NK frequency and function neither in responders nor in nonresponders. Among patients with undetectable HBV DNA levels at week 48, there were no significant differences between baseline and week 12 or 24 in terms of the frequencies of NK, CD56^bright^ NK, CD56^dim^ NK, IFNAR2^+^ NK, NKp46^+^ NK, NKp46^dim^ NK, and NKp46^bright^ NK cells. In addition, a similar trend was observed in the MFIs of IFNAR2 and NKp46 (Figure 6). In the nonresponder group, NK cell frequency was increased and the frequency of IFNAR2^+^ NK together with NKp46^bright^ NK cells as well as IFNAR2 MFI was decreased markedly from baseline to weeks 12 and 24 (Figure 6).

### 3.5. Comparison of the Effects of Interferon Therapy and Entecavir Therapy

We performed a chi-squared test to verify which treatment was the most effective through the evaluation of the patient number which had a positive response for PEG-IFN-*α* or entecavir treatment, respectively, by *Δ*HBsAg (log_10_) ≥ 1 or HBV DNA negative. Our study showed that the decrease of HBsAg in PEG-IFN group was more significant than that in the ETV group, as was shown in Table 3. However, in Table 3, for patients with HBV DNA negative, there was no significant difference between the ETV group and the PEG-IFN group.

## 4. Discussion

As HBV are not toxic to hepatocytes and the host's immune response contributes to liver inflammation and necrosis [24], it is necessary to gain better understanding on NK cells' role on mediating the innate immune responses during virus infection, as well as their role on regulating the adaptive immune responses in the persistent infection [11]. NK cells kill target cells via perforin/granzyme, Fas/Fas-L, TRAIL pathway, and ADCC. Activated NK cells secrete cytokines such as IFN-*γ* and TNF-*α* to enhance the body's anti-infective effect and participate in immune regulation [25]. In patients with chronic hepatitis B, during treatment, not only does PEG-IFN-*α* play a direct antiviral effect but it also stimulates immune cells, which results in removing virus-infected hepatocytes [24].

We found that in CHB patients, the numbers of CD3^−^CD56^+^ NK cells recovered after PEG-IFN therapy, accompanied by the normalization of ALT. This study indicated that NK cells are associated with liver injury. In 2015, Zheng et al. suggested that when the NK cell counts in peripheral blood decreased, the activated NK cells were enriched in the liver [2]. Furthermore, CD69^+^ NK cells were positively correlated with patient's ALT levels [2]. In the present study, we did not find that ALT levels were associated with changes in any of the frequencies of CD3^−^CD56^+^ NK, CD56^bright^ NK, CD56^dim^ NK, IFNAR2^+^ NK, NKp46^+^ NK, and NKp46^bright^ and NKp46^dim^ NK cells and in NKp46 MFI and IFNAR2 MFI.

Previous studies showed that the HBsAg and HBeAg levels in circulating peripheral blood could inhibit NK function, which led to insufficient immune without the clearance of HBV [24, 26, 27]. It suggested that an effective antiviral therapy should induce the recovery of NK function. It was reported that PEG-IFN-*α*-2a could significantly amplify CD56^bright^ NK cells and promote CD56^dim^ NK cell activation and enhance their function; at the same time, the absolute number of CD56^bright^ NK cells was negatively correlated with HBsAg titer [28, 29]. However, the details on dynamic changes of CD56^bright^ NK cells have not been explored in PEG-IFN versus entecavir therapy [30]. In our study, the frequency of CD56^bright^ NK and the expression of NK cell surface receptors NKp46 and IFNAR2 increased after PEG-IFN treatment, although the recovery of NK function was not detectable during early therapy of ETV in CHB patients while the frequency of NK cells increased obviously. Our results revealed that the decline of HBV DNA loads may be insufficient to recover the immune activity in oral antiviral therapy. IFN treatment could enhance immune control by increasing NK cells counts and enhancing recovery of NK cell function.

Our subgroup analyses further observed that during PEG-IFN-*α*-2a treatment, the expression of NKp46 and IFNAR2 on NK cells in nonresponse group had no significant differences compared with baseline, but in the response group, the expression of NKp46 and IFNAR2 on NK cells significantly enhanced, which suggested that the activation of NKp46 and IFNAR2 was related to the therapeutic effect. These results were consistent with previous findings [18, 31, 32] and further support the direction of using the aforementioned parameters as the early indicator to predict treatment response. This study suggested that NK cell function was not associated with changes in HBV DNA loads and HBeAg levels before treatment and during early treatment, but the HBsAg decrease was positively associated with NKp46^bright^ NK frequency before treatment and at treatment week 12. In clinical practice, the decrease in the HBsAg level is important for predicting the outcome of IFN-*α* therapy in CHB [33, 34]. However, during ETV therapy in CHB patients, the changes in HBsAg and HBeAg levels were not correlated to NK function, and the expression of NKp46 and IFNAR2 on NK cells in HBV DNA nonresponse patients significantly decreased at 12 and 48 weeks from baseline. These data may not verify the causal relationship between the virus and NK function. Our study demonstrated that, in both PEG-IFN-*α* and nucleoside analogue, antiviral therapeutic effects were correlated to the activation of NK cells.

Our study showed that the decrease of HBsAg in the PEG-IFN group was more significant than that in the ETV group, as was shown in Table 3. Studies showed that PEG-IFN focused on immune regulation, which made the decrease of HBsAg more advantageous [35–37]. However, studies demonstrated that ETV made HBV DNA negative more significantly than interferon [37, 38], but in Table 3, for patients with HBV DNA negative, there was no significant difference between the ETV group and PEG-IFN group, which may be related to the small sample size of our enrollment. Our study has some limitations, which included mainly those related to a single-center, limited sample size, and nonrandomized study. Further large clinical trials are warranted to verify our findings.

In addition, we need to add the following: NK cells were the important part of innate immunity. Interferon triggered innate immunity, which took effect at 12 weeks of treatment and peaks at 24 weeks. The early to midterm efficacy of interferon on natural killer cell induction determined the subsequent efficacy, so the changes in the characteristics of NK cells at 12 and 24 weeks of treatment were tested [10, 39, 40]. The patients were followed up for 48 weeks because the course of interferon treatment was 48 weeks, and patients treated with ETV as a control group were also followed up to 48 weeks.

## 5. Conclusions

In summary, in patients with chronic hepatitis B, PEG-IFN therapy had significant changes in NK cell frequencies and function when compared to entecavir-treated patients. The therapeutic response to PEG-IFN or entecavir during treatment was positively associated with NK cell function [41, 42]. Successful reduction of HBV DNA to the undetectable levels with entecavir was associated with enhance NK cell function. Our data imply that combined therapy with PEG-IFN and entecavir may be a promising option for patients with CHB. In addition, our findings point to the direction of using NK cell parameters as predictive markers of response in developing new therapeutic strategies for chronic hepatitis B patients.

## Figures and Tables

**Table 1:** Baseline clinical characteristics of chronic hepatitis B patients.

Characteristics	All patients *n* = 89	PEG-IFN group *n* = 49	ETV group *n* = 40	*P* value
Male, *n* (%)	59 (66%)	31 (63%)	28 (70%)	
Median Age (yrs) (Range)	31 (20–53)	30 (20–51)	32 (23–53)	0.115
Mean ± SD HBsAg level (log_10_ IU/mL)	3.94 ± 0.67	3.85 ± 0.72	4.05 ± 0.58	0.163
Mean ± SD HBV DNA load (log_10_ IU/mL)	7.31 ± 0.92	7.18 ± 1.03	7.45 ± 0.76	0.330
Median HBeAg concentration (PEIU/mL) (Q1, Q3)	902.26 (454.79, 1398.77)	878.35 (433.55, 1398.77)	1067.64 (490.87, 1400.37)	0.172
Median ALT level (U/L) (Q1, Q3)	238.80 (124.50, 344.60)	253.30 (129.85, 357.80)	223.15 (114.10, 343.93)	0.775

ALT: alanine aminotransferase; HBsAg: hepatitis B surface antigen; HBeAg: hepatitis B e antigen; *P* value: PEG-IFN group vs. ETV group.

**Table 2:** Changes of HBV markers during therapy.

Item	0 w			12 w			24 w			36 w			48 w		
	PEG-IFN group	ETV group	*P* value	PEG-IFN group	ETV group	*P* value	PEG-IFN group	ETV group	*P* value	PEG-IFN group	ETV group	*P* value	PEG-IFN group	ETV group	*P* value
Mean ± SD HBsAg level (log_10_ IU/mL)	3.85 ± 0.74	4.05 ± 0.58	0.280	3.04 ± 1.00	3.60 ± 0.93	0.004	2.79 ± 1.20	3.57 ± 0.75	0.003	2.66 ± 1.54	3.39 ± 1.20	0.040	2.65 ± 1.35	3.20 ± 0.73	0.132
Mean ± SD HBV DNA load (log_10_ IU/mL)	7.18 ± 1.00	7.44 ± 0.69	0.127	4.00 ± 1.60	2.80 ± 1.10	0.000	1.94 ± 0.83	2.18 ± 0.81	0.175	1.60 ± 0.71	1.89 ± 0.67	0.143	1.30 ± 0.95	1.59 ± 0.84	0.174
HBeAg Concentration (PEIU/mL) (Q1, Q3)	878.33 (432.55, 1397.77)	1066.62 (489.97, 1401.07)	0.472	52.07 (5.92, 381.71)	57.62 (13.91, 645.96)	0.155	17.02 (2.45, 70.52)	35.52 (6.48, 317.82)	0.039	13.31 (0.72, 49.70)	59.32 (4.51, 206.61)	0.622	7.65 (0.92, 25.32)	16.85 (2.60, 42.85)	0.575
Median ALT level (U/L) (Q1, Q3)	252.80 (128.95, 356.96)	222.45 (114.10, 344.03)	0.949	68.20 (49.45, 83.23)	33.30 (21.30, 49.80)	0.001	44.86 (25.87, 63.59)	23.12 (17.77, 32.89)	0.004	33.60 (28.15, 55.90)	22.80 (15.25, 25.85)	0.069	33.15 (17.34, 46.73)	20.12 (15.40, 28.98)	0.255

HBsAg: hepatitis B surface antigen; HBeAg: hepatitis B e antigen; ALT: alanine aminotransferase; *P* value: PEG-IFN group vs. ETV group.

**Table 3:** The chi-square test between the two groups by *Δ*HBsAg level (log_10_) or HBV DNA negative from 0 to 48 weeks.

Item	PEG-IFN group	ETV group	*χ* ^2^	*P* value
*Δ*HBsAg (log_10_) ≥ 1 (number)	31	10	12.979	0.000
*Δ*HBsAg (log_10_) < 1 (number)	18	30		
HBV DNA negative (number)	32	28	0.221	0.638
HBV DNA positive (number)	17	12		

HBsAg: hepatitis B surface antigen; *Δ*HBsAg (log_10_): the difference value of HBsAg (log_10_) from 0 week to 48 weeks; *P* value: PEG-IFN group vs. ETV group. *P* value of less than 0.05 was identified as statistically significant.

## Data Availability

If necessary, readers could contact our research group.

## Supplementary Materials

Supplementary Materials Supplementary Figure 1: using FlowJo software for CD3^−^CD56^+^ NK cell image analysis to illustrative dot plots related to the FACS data.
