# Supplementary material for: The Characteristics of Natural Killer Cells in Chronic Hepatitis B Patients Who Received PEGylated-Interferon versus Entecavir Therapy
Source: Biomed Res Int. 2021 Jan 25;2021:2178143. doi: 10.1155/2021/2178143 (PMC7857883; doi:10.1155/2021/2178143)
Supplement: Supplementary Materials — Supplementary Figure 1: using FlowJo software for CD3−CD56+ NK cell image analysis to illustrative dot plots related to the FACS data. [file 2178143.f1.doc]

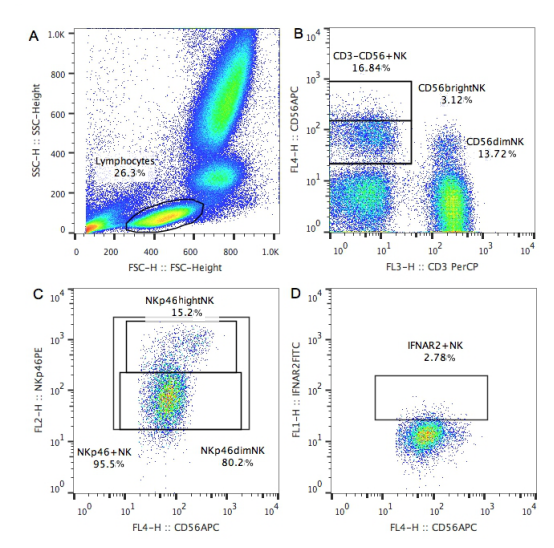


Supplementary figure.1. Using FlowJo software for CD3-CD56+NK cell image analysis to illustrative dot plots related to the FACS data.
